# The Laplacian-Energy-Like Invariants of Three Types of Lattices

**DOI:** 10.1155/2016/7320107

**Published:** 2016-03-30

**Authors:** Zheng-Qing Chu, Jia-Bao Liu, Xiao-Xin Li

**Affiliations:** ^1^Department of Public Courses, Anhui Xinhua University, Hefei, Anhui 230088, China; ^2^College of Mathematics and Computer, Chizhou University, Chizhou, Anhui 247000, China

## Abstract

This paper mainly studies the Laplacian-energy-like invariants of the modified hexagonal lattice, modified Union Jack lattice, and honeycomb lattice. By utilizing the tensor product of matrices and the diagonalization of block circulant matrices, we derive closed-form formulas expressing the Laplacian-energy-like invariants of these lattices. In addition, we obtain explicit asymptotic values of these invariants with software-aided computations of some integrals.

## 1. Introduction

Molecular structure descriptors or topological indices are used for modelling information of molecules, including toxicologic, chemical, and other properties of chemical compounds in theoretical chemistry. Topological indices play a very important role in mathematical chemistry, especially in the quantitative structure-property relationship (QSPR) and quantitative structure activity relationship (QSAR). Many topological indices have been introduced and investigated by mathematicians, chemists, and biologists, which contain energy [1], the Laplacian-energy-like invariant [2–5], the Kirchhoff index [6–13], and so forth. The energy of the graph is an important invariant of the adjacency spectrum and is the sum of the absolute values of all the eigenvalues of a graph *G*, which is studied in chemistry and used to approximate the total electron energy of a molecule [1]. During researching the character of the conjugated carbon oxides, chemists found that the “general electric” *E*_*π*_ is closely related to the energy releasing from the formation progress of the conjugated carbon oxides and could be approximately calculated by Hückel molecular orbital theory. And in the method of HMO, the calculation of *E*_*π*_ can be attributed to the sum of the absolute values of all the eigenvalues of its molecular graph [14–20].

Compared with adjacency matrix, the definition of Laplacian matrix added to all vertices degrees. As Mohar said, the Laplacian eigenvalues can reflect more the combination properties of graphs. Cvetković and Simić [21–23] pointed out that, as molecular structure descriptors, the Laplacian-energy-like invariant not only well describes the properties of most of the descriptors which are indicated, such as entropy, molar volume, and molar refractivity, but also is able to describe some more difficult properties, such as boiling point and rub points. Due to the fact that Laplacian-energy-like invariant has a significant physical and chemical background [24, 25], it has received wide attention to research it from many mathematical and chemical workers.

All the graphs discussed in this paper are simple, finite, and undirected. For a graph *G*, the vertex set and edge set of *G* will be denoted by *V*(*G*) = {*v*_1_, *v*_2_,…, *v*_*n*_} and *E*(*G*) = {*e*_1_, *e*_2_,…, *e*_*m*_}, respectively [26]. The adjacency matrix and the diagonal matrix of *G* are, respectively, *A*(*G*) and *D*(*G*); then the matrix *L*(*G*) = *D*(*G*) − *A*(*G*) is called the Laplacian matrix of the graph *G* [27, 28]. The characteristic polynomials and Laplacian polynomials of the graph *G* are *χ*_*G*_(*λ*) = det⁡(*λI* − *A*(*G*)) and *μ*_*G*_(*λ*) = det⁡(*λI* − *L*(*G*)) [29]. Both *A*(*G*) and *L*(*G*) are symmetric matrices; their eigenvalues are real numbers [30, 31]. Thus, we can order the eigenvalues of the graph *G* as *λ*_1_ ≥ *λ*_2_ ≥ ⋯≥*λ*_*n*_, and the Laplacian eigenvalues are *μ*_1_ ≥ *μ*_2_ ≥ ⋯≥*μ*_*n*_ [32, 33]. If *G* is a connected graph, then *μ*_*i*_ > 0, *i* = 1,2,…, *n* − 1, *μ*_*n*_ = 0  [34–36]. Next, we will recall some basic concepts.

**Definition 1 (see [1]).**

The energy of a graph *G* is the sum of the absolute values of all the eigenvalues of *G*; that is,

(1) $$\begin{array}{c} E\left(\;G\;\right)=\overset{n}{\underset{i=1}{\sum }}\left|\;\lambda_{i}\;\right|. \end{array}$$

**Definition 2 (see [2]).**

Let *G* be a graph of order *n*. The Laplacian-energy-like invariant of *G*, denoted by LEL(*G*), is defined as

(2) $$\begin{array}{c} \text{LEL}\left(\;G\;\right)=\overset{n}{\underset{i=1}{\sum }}\sqrt{\mu_{i}}. \end{array}$$

**Definition 3 (see [35]).**

For two matrices *A* = (*a*_*i*,*j*_)_*m*×*n*_, *B* = (*b*_*i*,*j*_)_*s*×*t*_, the tensor product of *A* and *B*, denoted by *A* ⊗ *B*, is defined as

(3) $$\begin{array}{c} \left(\begin{array}{cccc} a_{11}B & a_{12}B & \cdots & a_{1n}B\\ \cdots & \cdots & \cdots & \cdots \\ \cdots & \cdots & \cdots & \cdots \\ a_{m1}B & a_{m2}B & \cdots & a_{mn}B \end{array}\right). \end{array}$$

**Theorem 4 (see [35]).**

Let {*G*_*n*_} be a sequence of finite simple graphs with bounded average degree such that

(4) limn→∞⁡VGn=∞,limn→∞⁡LELGnVGn=h≠0.

Let {*H*_*n*_} be a sequence of spanning subgraphs of {*G*_*n*_} such that

(5) $$\begin{array}{c} \underset{n\to \infty }{\lim}\frac{\left|\left\{v\in V\left(H_{n}\right):d_{H_{n}\left(v\right)}=d_{G_{n}\left(v\right)}\right\}\right|}{\left|V\left(G_{n}\right)\right|}=1; \end{array}$$

then

(6) $$\begin{array}{c} \underset{n\to \infty }{\lim}\frac{LEL\left(H_{n}\right)}{\left|V\left(G_{n}\right)\right|}=h. \end{array}$$

That is, *G*_*n*_ and *H*_*n*_ have the same asymptotic Laplacian-energy-like invariant.

In what follows, we will explore the Laplacian-energy-like invariants formulas of the modified hexagonal lattice, modified Union Jack lattice, and honeycomb lattice.

## 2. Main Results

### 2.1. The Laplacian-Energy-Like Invariant of the Modified Hexagonal Lattice

The modified hexagon lattice with toroidal boundary condition is denoted by MH^*t*^(*n*_1_, *n*_2_).

**Theorem 5 .**

Let *α*_*i*_ = 2*πi*/*n*_1_, *β*_*j*_ = 2*πj*/*n*_2_. Then

(7) 1  LELMHtn1,n2=∑i=0n1−1 ∑j=0n2−16−2cos⁡αi−2cos⁡βj−2cos⁡αi−βj,2  limn1→∞⁡ limn2→∞⁡LELMHtn1,n2n1n2=14π2·∬02π6−2cos⁡x−2cos⁡y−2cos⁡x−ydx dy≈2.3705.

**Proof**

With the proper labelling of the vertices of the modified hexagonal lattice, its Laplacian matrix is

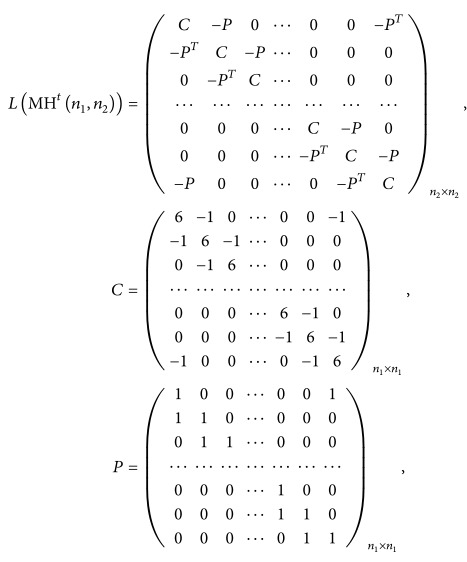
(8)where *I*_*n*_1__, *I*_*n*_2__ are the unit matrices and *M* ⊗ *N* is tensor product of matrices *M* and *N*. Consider

(9) $$\begin{array}{c} B_{n_{2}}={\left(\;\begin{array}{cccccc} 0 & 1 & 0 & \cdots & 0 & 0\\ 0 & 0 & 1 & \cdots & 0 & 0\\ \cdots & \cdots & \cdots & \cdots & \cdots & \cdots \\ 0 & 0 & 0 & \cdots & 0 & 1\\ 1 & 0 & 0 & \cdots & 0 & 0 \end{array}\;\right)}_{n_{2}\times n_{2}}. \end{array}$$

The matrix *L*(MH^*t*^(*n*_1_, *n*_2_)) can be defined as follows:

(10) LMHtn1,n2In2⊗Cn1−Bn2⊗Pn1−Bn2T⊗Pn1T=In2⊗6In1−Bn1−Bn1T−Bn2⊗In1+Bn1T−Bn2T⊗In1+Bn1.

Let {1 = *g* ^0^, *g* ^1^,…, *g* ^*n*−1^} be a cyclic group of order *n*. Obviously, *ρ* : *g* ^*i*^ → *B*_*n*_^*i*^ can express the group. The cyclic group of order *n* has linear values of *n* *χ*_*i*_  (*i* = 0,1,…, *n* − 1),  *χ*_*i*_(*g*) = *ω*_*n*_^*i*^, where *ω*_*n*_ are said *n*-times unit roots.

Therefore, there is a reversible matrix

(11) $$\begin{array}{c} Q_{n}={\left(\;\frac{\omega_{n}^{ij}}{\sqrt{n}}\;\right)}_{0\leq i,j\leq n-1}, \end{array}$$

such that

(12) $$\begin{array}{c} Q_{n}^{-1}B_{n}Q_{n}=\mathrm{diag}\left(\;1,\omega_{n},\ldots ,\omega_{n}^{n-1}\;\right)≕D_{n}. \end{array}$$

In fact,

(13) BnT=Bn−1,QnT=Qn−1;

hence

(14) $$\begin{array}{c} Q_{n}^{-1}B_{n}^{T}Q_{n}=\mathrm{diag}\left(\;1,\omega^{-1},\ldots ,\omega_{n}^{-\left(n-1\right)}\;\right)≕D_{n}^{-1}. \end{array}$$

So

(15) Qn2−1⊗Qn1−1LMHtn1,n2Qn2⊗Qn1=Qn2−1⊗Qn1−1In2⊗6In1−Bn1−Bn1T−Bn2⊗In1+Bn1T−Bn2T⊗In1+Bn1Qn2⊗Qn1=In2⊗6In1−Dn1−Dn1−1−Dn2⊗In1+Dn1−1−Dn2−1⊗In1+Dn1.

It is not difficult to find that *I*_*n*_2__ ⊗ (6*I*_*n*_1__ − *D*_*n*_1__ − *D*_*n*_1__^−1^) − *D*_*n*_2__ ⊗ (*I*_*n*_1__ + *D*_*n*_1__^−1^) − *D*_*n*_2__^−1^ ⊗ (*I*_*n*_1__ + *D*_*n*_1__) is a diagonal matrix whose diagonal elements are

(16) 6−ωn1i−ωn1−i−ωn2j−ωn2−j−ωn1iωn2−j+ωn1−iωn2j=6−2cos⁡2iπn1−2cos⁡2jπn2−2cos⁡2iπn1−cos⁡2jπn2,

where 0 ≤ *i* ≤ *n*_1_ − 1 and  0 ≤ *j* ≤ *n*_2_ − 1.

This means that the eigenvalues of the matrix *L* are *μ* = 6 − 2cos⁡*α*_*i*_ − 2cos⁡*β*_*j*_ − 2cos⁡(*α*_*i*_ − *β*_*j*_), 0 ≤ *i* ≤ *n*_1_ − 1, and  0 ≤ *j* ≤ *n*_2_ − 1, where *α*_*i*_ = 2*πi*/*n*_1_ and *β*_*j*_ = 2*πj*/*n*_2_.

By formula (2), the Laplacian-energy-like invariant is

(17) LELMHtn1,n2=∑i=0n1−1 ∑j=0n2−16−2cos⁡αi−2cos⁡βj−2cos⁡αi−βj.

So

(18) limn1→∞⁡ limn2→∞⁡LELMHtn1,n2n1n2=limn1→∞ limn2→∞⁡1n1n2·∑i=0n1−1 ∑j=0n2−16−2cos⁡αi−2cos⁡βj−2cos⁡αi−βj=∬016−2cos⁡2πx−2cos⁡2πy−2cos⁡2πx−ydx dy=14π2∬02π6−2cos⁡x−2cos⁡y−2cos⁡x−ydx dy≈2.3705.

**Remark 6 .**

The numerical integration value in last line is calculated with the software MATLAB [37]. As such computations would be possible on a computer with high memory and processing speed, we used Mac Pro with processor 2 × 2.93 GHz 6-core Intel Xeon (24 hyperthreads in total) and memory 24 GB 1333 MHz DDR3 to obtain the results.

By Theorems 4 and 5, we can immediately arrive at the following theorem.

**Theorem 7 .**

For the modified hexagonal lattices *MH* ^*t*^(*n*_1_, *n*_2_), *MH* ^*c*^(*n*_1_, *n*_2_), and *MH* ^*f*^(*n*_1_, *n*_2_) with toroidal, cylindrical, and free boundary conditions, then,

(19) 1  limn1→∞⁡ limn2→∞⁡LELMHtn1,n2n1n2=limn1→∞⁡ limn2→∞⁡LELMHcn1,n2n1n2=limn1→∞⁡ limn2→∞⁡LELMHfn1,n2n1n2≈2.3705,2  LELMHtn1,n2=LELMHcn1,n2=LELMHfn1,n2≈2.3705n1n2.

### 2.2. The Laplacian-Energy-Like Invariant of the Modified Union Jack Lattice

The modified Union Jack lattice with toroidal boundary condition is denoted by *S* ^*t*^(*n*_1_, *n*_2_).

**Theorem 8 .**

Let *α*_*i*_ = 2*πi*/*n*_1_;  *β*_*j*_ = 2*πj*/*n*_2_. Then

(20) 1  LELStn1,n2=∑i=0n1−1 ∑j=0n2−18−2cos⁡αi−2cos⁡βj−4cos⁡αicos⁡βj,2  limn1→∞⁡ limn2→∞⁡LELStn1,n2n1n2=14π2·∬02π8−2cos⁡x−2cos⁡y−4cos⁡xcos⁡y dx dy≈2.7586.

**Proof**

With a proper labelling of the vertices of the modified Union Jack lattice, its Laplacian matrix can be represented as

(21) LStn1,n2=G−U0⋯00−UT−UTG−U⋯0000−UTG⋯000⋯⋯⋯⋯⋯⋯⋯000⋯G−U0000⋯−UTG−U−U00⋯0−UTGn2×n2,G=8−10⋯00−1−18−1⋯0000−18⋯000⋯⋯⋯⋯⋯⋯⋯000⋯8−10000⋯−18−1−100⋯0−18n1×n1,U=110⋯001111⋯000011⋯000⋯⋯⋯⋯⋯⋯⋯000⋯110000⋯111100⋯011n1×n1.

Based on Theorem 5, we get

(22) LStn1,n2In2⊗Gn1−Bn2⊗Un1−Bn2T⊗Un1T=In2⊗8In1−Bn1−Bn1T−Bn2⊗In1+Bn1+Bn1T−Bn2T⊗In1+Bn1T+Bn1.

Let

(23) $$\begin{array}{c} Q_{n}={\left(\;\frac{\omega_{n}^{ij}}{\sqrt{n}}\;\right)}_{0\leq i,j\leq n-1}, \end{array}$$

such that

(24) $$\begin{array}{c} Q_{n}^{-1}B_{n}Q_{n}=\mathrm{diag}\left(\;1,\omega_{n},\ldots ,\omega_{n}^{n-1}\;\right)≕D_{n}. \end{array}$$

Actually,

(25) BnT=Bn−1,QnT=Qn−1;

consequently,

(26) $$\begin{array}{c} Q_{n}^{-1}B_{n}^{T}Q_{n}=\mathrm{diag}\left(\;1,\omega^{-1},\ldots ,\omega_{n}^{-\left(n-1\right)}\;\right)≕D_{n}^{-1}. \end{array}$$

So

(27) Qn2−1⊗Qn1−1LStn1,n2Qn2⊗Qn1=Qn2−1⊗Qn1−1In2⊗8In1−Bn1−Bn1T−Bn2⊗In1+Bn1+Bn1T−Bn2T⊗In1+Bn1T+Bn1Qn2⊗Qn1=In2⊗8In1−Dn1−Dn1−1−Dn2⊗In1+Dn1+Dn1−1−Dn2−1⊗In1+Dn1−1+Dn1.

It is not difficult to find that

(28) In2⊗8In1−Dn1−Dn1−1−Dn2⊗In1+Dn1+Dn1−1−Dn2−1⊗In1+Dn1−1+Dn1

is a diagonal matrix whose diagonal elements are

(29) 8−ωn1i−ωn1−i−ωn2j−ωn2−j−ωn1iωn2j+ωn1iωn2−j+ωn1−iωn2−j+ωn1−iωn2j=8−2cos⁡2iπn1−2cos⁡2jπn2−4cos⁡2iπn1cos⁡2jπn2,

where 0 ≤ *i* ≤ *n*_1_ − 1 and  0 ≤ *j* ≤ *n*_2_ − 1.

This means that the eigenvalues of the matrix *L* are *μ* = 8 − 2cos⁡*α*_*i*_ − 2cos⁡*β*_*j*_ − 4cos⁡*α*_*i*_ *β*_*j*_, 0 ≤ *i* ≤ *n*_1_ − 1, and  0 ≤ *j* ≤ *n*_2_ − 1, where *α*_*i*_ = 2*πi*/*n*_1_ and *β*_*j*_ = 2*πj*/*n*_2_.

By formula (2), the Laplacian-energy-like invariant is

(30) LELStn1,n2=∑i=0n1−1 ∑j=0n2−18−2cos⁡αi−2cos⁡βj−4cos⁡αicos⁡βj.

So

(31) limn1→∞⁡ limn2→∞⁡LELStn1,n2n1n2=limn1→∞⁡ limn2→∞⁡1n1n2·∑i=0n1−1 ∑j=0n2−18−2cos⁡αi−2cos⁡βj−4cos⁡αicos⁡βj=∬018−2cos⁡2πx−2cos⁡2πy−4cos⁡2πxcos⁡2πy dx dy=14π2∬02π8−2cos⁡x−2cos⁡y−4cos⁡xcos⁡y dx dy≈2.7586.

By Theorems 4 and 8, it is not difficult to arrive at the following theorem.

**Theorem 9 .**

For the modified Union Jack lattices *S* ^*t*^(*n*_1_, *n*_2_), *S* ^*c*^(*n*_1_, *n*_2_), and *S* ^*f*^(*n*_1_, *n*_2_) with toroidal, cylindrical, and free boundary conditions, then,

(32) 1  limn1→∞⁡ limn2→∞⁡LELStn1,n2n1n2=limn1→∞⁡ limn2→∞⁡LELScn1,n2n1n2=limn1→∞⁡ limn2→∞⁡LELSfn1,n2n1n2≈2.7586,2  LELStn1,n2=LELScn1,n2=LELSfn1,n2≈2.7586n1n2.

### 2.3. The Laplacian-Energy-Like Invariant of the Honeycomb Lattice

The honeycomb lattice with toroidal boundary condition, denoted by HC^*t*^(*n*_1_, *n*_2_), can be constructed by starting with an *m* × *n* square lattice and adding two diagonal edges to each square.

**Theorem 10 .**

Let *α*_*i*_ = 2*πi*/*n*_1_ and *β*_*j*_ = 2*πj*/*n*_2_. Then

(33) 1  LELHCtn1,n2=∑i=0n1−1 ∑j=0n2−13+3+2cos⁡αi+2cos⁡βj+2cos⁡αi−βj+∑i=0n1−1 ∑j=0n2−13−3+2cos⁡αi+2cos⁡βj+2cos⁡αi−βj,2  limn1→∞⁡ limn2→∞⁡LELHCtn1,n22n1n2=18π2·∬02π3+3+2cos⁡x+2cos⁡y+2cos⁡x−ydx dy+18π2·∬02π3−3+2cos⁡x+2cos⁡y+2cos⁡x−ydx dy≈1.6357.

**Proof**

Similarly, the Laplacian matrix of the honeycomb lattice is $L\left({\text{HC}}^{t}\left(n_{1},n_{2}\right)\right)=\left(\begin{array}{cc} 3I_{M} & -F\\ -F^{T} & 3I_{M} \end{array}\right)$, where *M* = *n*_1_ *n*_2_ and *F* is an *M* × *M* matrix. The matrix *F* can be written in the following form:

(34) F=W00⋯0IIIW0⋯0000IW⋯000⋯⋯⋯⋯⋯⋯⋯000⋯W00000⋯IW0000⋯0IWn2×n2,W=100⋯001110⋯000011⋯000⋯⋯⋯⋯⋯⋯⋯000⋯100000⋯110100⋯011n1×n1,

where *I* represents the unit matrix of *n*_1_ × *n*_1_ and *I*_*M*_ represents the unit matrix of *M* × *M*, respectively.

Based on Theorem 5, the matrix *F* can be written as

(35) FIn2⊗Wn1+Bn2T⊗In1=In2⊗In1+Bn1T+Bn2T⊗In1.

Let

(36) $$\begin{array}{c} Q_{n}={\left(\;\frac{\omega_{n}^{ij}}{\sqrt{n}}\;\right)}_{0\leq i,j\leq n-1}, \end{array}$$

such that

(37) $$\begin{array}{c} Q_{n}^{-1}B_{n}Q_{n}=\mathrm{diag}\left(\;1,\omega_{n},\ldots ,\omega_{n}^{n-1}\;\right)≕D_{n}. \end{array}$$

Similarly,

(38) BnT=Bn−1,QnT=Qn−1;

hence,

(39) $$\begin{array}{c} Q_{n}^{-1}B_{n}^{T}Q_{n}=\mathrm{diag}\left(\;1,\omega^{-1},\ldots ,\omega_{n}^{-\left(n-1\right)}\;\right)≕D_{n}^{-1}. \end{array}$$

So

(40) Qn2−1⊗Qn1−1FQn2⊗Qn1=Qn2−1⊗Qn1−1·In2⊗In1+Bn1T+Bn2T⊗In1Qn2⊗Qn1=In2⊗In1+Dn1−1+Dn2−1⊗In1.

It is not difficult to find that *I*_*n*_2__ ⊗ (*I*_*n*_1__ + *D*_*n*_1__^−1^) + *D*_*n*_2__^−1^ ⊗ *I*_*n*_1__ is a diagonal matrix whose diagonal elements are 1 + *ω*_*n*_1__^−*i*^ + *ω*_*n*_2__^−*j*^, so matrix *L*(HC^*t*^(*n*_1_, *n*_2_)) can be reduced to the following form:

(41) LHCtn1,n2=3−1−ωn1−i−ωn2−j−1−ωn1−i−ωn2−j3.

By det⁡(*μI* − *L*(HC^*t*^(*n*_1_, *n*_2_))) = 0, we can get

(42) μ−32−1−ωn1−i−ωn2−j−1−ωn1−i−ωn2−j=3+ωn1i+ωn1−i+ωn2j+ωn2−j+ωn1−iωn2j+ωn1iωn2−j=3+2cos⁡2πin1+2cos⁡2πjn2+2cos⁡cos⁡2πin1−cos⁡2πjn2.

Therefore, the *L*(HC^*t*^(*n*_1_, *n*_2_)) characteristic eigenvalues are

(43) μ=3±3+2cos⁡2πin1+2cos⁡2πjn2+2cos⁡2πin1−2πjn2,

where 0 ≤ *i* ≤ *n*_1_ − 1 and 0 ≤ *j* ≤ *n*_2_ − 1.

Let *α*_*i*_ = 2*πi*/*n*_1_ and *β*_*j*_ = 2*πj*/*n*_2_. By formula (2), we may obtain the Laplacian-energy-like invariant:

(44) LELHCtn1,n2=∑i=0n1−1 ∑j=0n2−13+3+2cos⁡αi+2cos⁡βj+2cos⁡αi−βj+∑i=0n1−1 ∑j=0n2−13−3+2cos⁡αi+2cos⁡βj+2cos⁡αi−βj.

By the definition of double integration, we arrive at

(45) limn1→∞⁡ limn2→∞⁡LELHCtn1,n22n1n2limn1→∞⁡ limn2→∞⁡12n1n2∑i=0n1−1 ∑j=0n2−13+3+2cos⁡αi+2cos⁡βj+2cos⁡αi−βj+limn1→∞⁡ limn2→∞⁡12n1n2∑i=0n1−1 ∑j=0n2−13−3+2cos⁡αi+2cos⁡βj+2cos⁡αi−βj=12∬013+3+2cos⁡2πx+2cos⁡2πy+2cos⁡2πx−ydx dy+12∬013−3+2cos⁡2πx+2cos⁡2πy+2cos⁡2πx−ydx dy=18π2∬02π3+3+2cos⁡x+2cos⁡y+2cos⁡x−ydx dy+18π2∬02π3−3+2cos⁡x+2cos⁡y+2cos⁡x−ydx dy≈1.6357.

By Theorems 4 and 10, we can easily obtain the following theorem.

**Theorem 11 .**

For the honeycomb lattices HC^*t*^(*n*_1_, *n*_2_), HC^*c*^(*n*_1_, *n*_2_), and HC^*f*^(*n*_1_, *n*_2_) with toroidal, cylindrical, and free boundary conditions, then,

(46) 1  limn1→∞⁡ limn2→∞⁡LELHCtn1,n2n1n2=limn1→∞⁡ limn2→∞⁡LELHCcn1,n2n1n2=limn1→∞⁡ limn2→∞⁡LELHCfn1,n2n1n2≈1.6357;2  LELHCtn1,n2=LELHCcn1,n2=LELHCfn1,n2≈1.6357n1n2.

## 3. Conclusions

In this paper, we mainly studied the Laplacian-energy-like invariants of the modified hexagonal lattice, modified Jack lattice, and honeycomb lattice. The Laplacian-energy-like invariants formulas of these lattices are obtained. The proposed results imply that the asymptotic Laplacian-energy-like invariants of those lattices are independent of the three boundary conditions.

The problems on the various topological indices of lattices have much important significance in the mathematical theory, chemical energy, statistical physics, and networks science. This paper investigated the Laplacian-energy-like invariants of some lattices. However, the other topological indices of the general lattices remain to be studied.
